# Polyamines: the pivotal amines in influencing the tumor microenvironment

**DOI:** 10.1007/s12672-024-01034-9

**Published:** 2024-05-18

**Authors:** Cassandra E. Holbert, Robert A. Casero, Tracy Murray Stewart

**Affiliations:** Sidney Kimmel Comprehensive Cancer Center, Johns Hopkins School of Medicine, Baltimore, MD USA

**Keywords:** Polyamine, Tumor microenvironment, Amino acid metabolism, Immunotherapy

## Abstract

Cellular proliferation, function and survival is reliant upon maintaining appropriate intracellular polyamine levels. Due to increased metabolic needs, cancer cells elevate their polyamine pools through coordinated metabolism and uptake. High levels of polyamines have been linked to more immunosuppressive tumor microenvironments (TME) as polyamines support the growth and function of many immunosuppressive cell types such as MDSCs, macrophages and regulatory T-cells. As cancer cells and other pro-tumorigenic cell types are highly dependent on polyamines for survival, pharmacological modulation of polyamine metabolism is a promising cancer therapeutic strategy. This review covers the roles of polyamines in various cell types of the TME including both immune and stromal cells, as well as how competition for nutrients, namely polyamine precursors, influences the cellular landscape of the TME. It also details the use of polyamines as biomarkers and the ways in which polyamine depletion can increase the immunogenicity of the TME and reprogram tumors to become more responsive to immunotherapy.

## Introduction

Polyamines are small polycationic molecules that are protonated at physiologic pH and interact with a variety of negatively charged macromolecules [1]. These interactions contribute to the molecular function of polyamines, including roles in chromatin remodeling, immune cell modulation, ion channel regulation, cellular survival and proliferation [2, 3]. All cell types require polyamines for normal function; however, cancer cells require elevated polyamine levels to support their continual proliferation. Elevated polyamine levels in tumor cells are maintained through dysregulated metabolism and increased uptake from the extracellular environment [4, 5]. Increased uptake by tumors decreases the availability of polyamines and their metabolic precursors to nearby normal cells and dampens their function. As polyamine metabolism is tied to the expression of numerous oncogenes and many tumors are reliant upon elevated polyamine levels, modulating polyamine metabolism is of particular interest as a cancer therapeutic [6–11]. In addition to promoting tumor survival, polyamines are known to encourage a tumor-permissive microenvironment through a multitude of mechanisms [12–22]. This review aims to examine the influence of polyamines on various cell types within the tumor microenvironment (TME), the impact of polyamines on the landscape of the TME and to discuss current attempts to modulate the TME to be less tumor-permissive by polyamine depletion.

## Polyamine metabolism

**Fig. 1:**
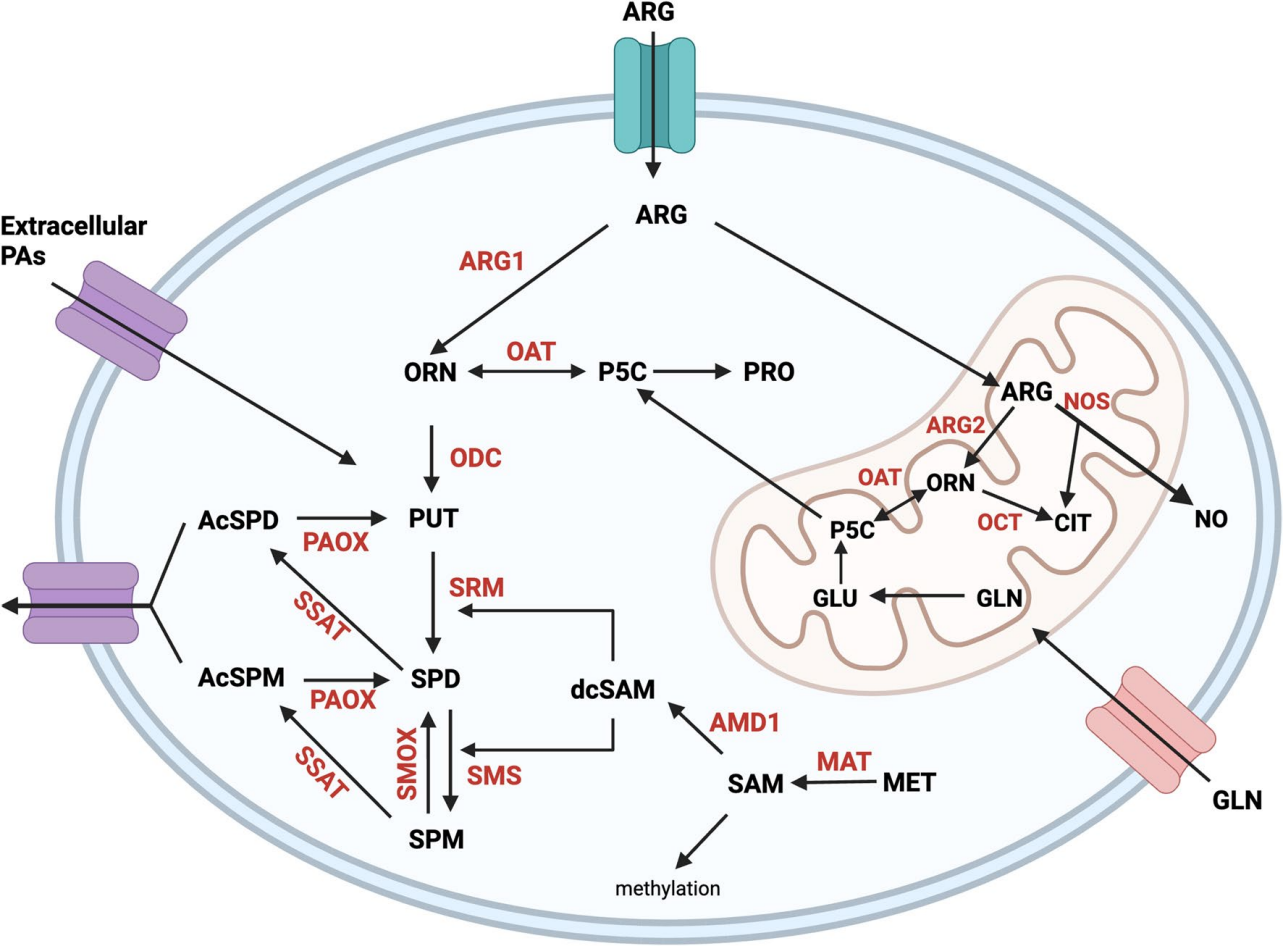
Polyamine metabolism is intrinsically linked with arginine, glutamine and methionine metabolism. Arginine (ARG) is catabolized by arginase 1 (ARG1) to form ornithine (ORN). Ornithine then feeds into polyamine metabolism by being decarboxylated by ornithine decarboxylase (ODC) to form putrescine (PUT). PUT uses decarboxylated *S*-adenosylmethionine (dcSAM) as an aminopropyl donor to form spermidine (SPD). Methionine adenosyltransferase (MAT) acts on methionine (MET) to form *S*-adenosylmethionine (SAM), which is subsequently decarboxylated by *S*-adenosylmethionine decarboxylase (AMD1). dcSAM is also the aminopropyl donor for conversion of SPD into spermine (SPM). SPM and SPD can be acetylated by spermidine/spermine *N*^1^-acetyltransferase (SSAT). *N*^1^*-*acetylated spermine or spermidine (AcSPM, AcSPD) can either be exported from the cell or further oxidized by polyamine oxidase (PAOX) to form SPD and PUT, respectively. ORN can alternatively be converted to proline (PRO) by way of a pyrroline-5-carboxylate (P5C) intermediate formed by ornithine aminotransferase (OAT) activity. Within the mitochondria, ARG can be metabolized to ORN by arginase 2 (ARG2) or to nitric oxide (NO) and citrulline (CIT) by nitric oxide synthase (NOS). CIT can also be formed from ORN by ornithine transcarbamylase (OTC). Glutamine (GLN) is metabolized to glutamate (GLU) and can serve as an alternative precursor to ornithine through P5C as an intermediate. Figure created using BioRender.com

Polyamine metabolism is regulated through coordinated biosynthesis, catabolism, and transport (Fig. 1). The polyamine precursor, l-ornithine, is decarboxylated into putrescine by one of the rate-limiting enzymes in polyamine biosynthesis, ornithine decarboxylase (ODC). Notably, ODC transcription is directly controlled by MYC; therefore, upregulation or amplification of *MYC* leads to increased polyamine biosynthesis in malignant tumors [6]. An aminopropyl group is added to putrescine by spermidine synthase (SRM) to produce spermidine, and spermidine can subsequently be transformed into spermine by the addition of a second aminopropyl group by spermine synthase (SMS) [4]. The aminopropyl groups added to the polyamines are derived from the decarboxylation of *S*-adenosylmethionine (SAM) by the second rate-limiting enzyme of polyamine biosynthesis, *S*-adenosylmethionine decarboxylase (AMD1). Importantly, once SAM has been decarboxylated to serve as the aminopropyl donor in polyamine biosynthesis, it is no longer available to be used as a methyl donor in transmethylation reactions [1]. Elevated decarboxylated SAM levels result in decreased activity of DNA methyltransferases and can result in dysregulated global methylation status and global transcriptional changes [23–26]. As such, polyamines are implicated in the epigenetic regulation of both ageing and cancer development and survival.

Catabolism of the higher order polyamines begins with spermidine/spermine *N*^1^-acetyltransferase (SSAT), which transfers an acetyl group from acetyl CoA to the *N*^1^ position of either spermidine or spermine [4, 27, 28]. *N*^1^-acetylated spermidine and spermine are predominantly exported through a polyamine transporter, explored in other reviews, or can be oxidized by the peroxisomal enzyme, acetylpolyamine oxidase (PAOX) to yield spermidine or putrescine [29–32]. The oxidation by PAOX produces toxic byproducts including 3-acetoamidopropanal (3-AAP) and H_2_O_2_, a precursor for reactive oxygen species. Polyamine catabolism must be tightly regulated as persistent exposure to these toxic byproducts can lead to oxidative stress, DNA damage and eventual apoptosis [33, 34].

Spermine can also be directly catabolized to spermidine without an acetylated intermediary by spermine oxidase (SMOX). The byproducts of SMOX activity include H_2_O_2_ and the lysosomotropic aldehyde 3-aminopropanal (3-AP) [35]. 3-AP causes oxidative stress and apoptosis by rupturing lysosomes, but can also spontaneously convert into the highly reactive and broadly toxic aldehyde, acrolein [36–39]. Importantly, SMOX is active in both the cytoplasmic and the nuclear compartments of the cell, and extensive SMOX activity in the latter results in decreased spermine, which can function as an antioxidant, as well as ROS production in close proximity to DNA [40–42]. While this can play a potentially damaging role in normal cells as a jump-start for inflammation-associated carcinogenesis, polyamine catabolism-induced oxidative damage can be utilized as a tumoricidal tactic in already transformed cells [16, 17, 43–47].

## Precursors and other metabolites of ornithine

### Arginine

Arginine metabolism occurs through the urea cycle, which includes the conversion of arginine to ornithine, citrulline and urea (Fig. 1). The production of ornithine involves the enzyme arginase, however arginine can alternatively be converted to nitric oxide (NO) by NO synthase (NOS). The interplay between arginase and NOS activity is a chief determinant in macrophage polarization and will be discussed later in this review. High arginase activity is indicative of poor outcome and is positively correlated with *MYCN* amplification, particularly in neuroblastoma, bladder, and ovarian cancers [48–50]. Chalishazar and colleagues have shown that MYC-driven small-cell lung cancer depends on arginine and polyamine levels for growth, and that depletion of arginine suppresses tumor growth and promotes the survival of mice with MYC-driven tumors [51]. Increased arginase activity has been detected in patients with lung, breast, prostate, and colon cancer and is hypothesized to be a mechanism of sustaining the polyamine levels required for tumor growth [52, 53]. It is important to note that this study by Chalishazar et al. was completed using patient-derived xenograft models in severely immunocompromised NSG mice [51]. As discussed later in this review, arginine is also required for adequate T-cell function. This paradox presents the possibility that while arginine depletion may suppress tumor growth, it may also dampen immune function thereby increasing tumor progression. Further studies evaluating arginine depletion in immunocompetent models are necessary to adequately determine the role of depletion on tumor progression.

### Glutamine

While arginine is the primary source of de novo ornithine synthesis in adult tissues, glutamine can be used as an alternative precursor to ornithine [54]. Within the mitochondria, glutamine is degraded to glutamate, which can be subsequently transformed into ornithine by the reversable enzyme ornithine δ-aminotransferase (OAT). A recent groundbreaking article by Lee et al. discovered that pancreatic adenocarcinoma (PDA) cells use glutamine as the preferred carbon source for de novo ornithine rather than arginine [55]. Lee and colleagues propose that PDA depends on glutamine for de novo polyamine synthesis due to both its oncogenic driver KRAS and arginine depletion within in its TME [55]. This suggests that the oncogenic drivers may influence the pathway for de novo polyamine synthesis, with MYC-driven cancers potentially preferring arginine as a precursor and KRAS-driven cancers preferring glutamine. Importantly, other cells of the TME, as discussed later in this review, require both arginine and glutamine for their function. At different points in tumor progression, these cells may have varying dependencies on each substrate, altering their availability for tumor cells.

### Proline

The activity of OAT is reversible, and the enzyme can use ornithine as a substrate to catalyze the transfer of the δ-amino group from ornithine to α-ketoglutarate to produce the end products of glutamate and l-pyrroline-5-carboxylate (P5C) [56]. l-P5C is then rapidly converted into l-proline [57]. As such, proline can be synthesized from either glutamate or ornithine with both routes converging at P5C. Proline is the second most common amino acid in collagen [58]. Ornithine increases extracellular pools of proline in wounds to increase collagen production [59]. ALDH18A1, the gene encoding pyrroline-5-carboxylate synthase (P5CS), has been implicated in both breast cancer and melanoma, and mutations in ALDH18A1 can cause cutaneous phenotypes including loose skin with low elasticity [60–63]. A recent characterization of a novel ALDH18A1 mutation found that patient fibroblasts show a reduction in proline, glutathione and putrescine production alongside abundant transcriptional changes in extracellular matrix-related genes [64]. Oral administration of l-ornithine increases collagen and polyamines in mouse skin, while supplementation of arginine results in increased ornithine and polyamines leading to an increase in collagen secretion by corneal fibroblasts [65, 66].

### Citrulline

Excess ornithine can be converted by ornithine transcarbamylase (OTC) to citrulline, a non-proteinogenic amino acid. Through the activity of NOS, arginine can also be converted to citrulline as an alternative to ornithine [67]. While more research is needed, upregulation of citrullination has been linked with a repression of epithelial-to-mesenchymal transition (EMT) in lung cancer cell lines [68].

## Polyamines and cells of the TME

Polyamines are required for the growth and function of all cells in the TME, including tumor cells, immune cells and stromal cells. Notably, polyamines are required for the development and activation of T-cells; however, tumor cells and immunosuppressive cells tend to deplete the TME of available polyamines and polyamine precursors, thereby dampening the function of T-cells. This is because polyamines are required for the immunosuppressive functions of tumor-associated macrophages and myeloid-derived suppressor cells. The influence of polyamines on many other cell types, particularly in immune cell subsets, has been well-covered in recent reviews [69–73]. Polyamines are also involved in the survival and function of stromal cells, including cancer-associated fibroblasts and endothelial cells.

### T-cells

Tumor-infiltrating lymphocytes (TILs) play a pivotal role in the immunogenicity of the TME. T-cells are typically the major component of TILs within the TME, namely CD4^+^ helper T-cells, CD8^+^ cytotoxic T-cells, and CD25^+^ regulatory T-cells (Tregs) [74, 75]. Polyamines play a role in numerous areas of the adaptive immune system, including B-cell lymphopoiesis, B-cell activation, and T-cell development and have been discussed in a previous review [73]. Polyamines are instrumental in normal T-cell function and survival [76–78]. T-cells upregulate their polyamine biosynthesis as well as their uptake of polyamines from their environment. Activated T-cells import more polyamines than naïve T-cells and prefer arginine as their major carbon donor for polyamine synthesis [79]. Arginine, ornithine, and polyamines are required for T-cell activation and signaling events by T-cell receptors (TCR) [80, 81]. TCR activation in CD4^+^ T-cells is mediated by the conversion of arginine into ornithine, and the proliferation and activity of T-cells following TCR activation is fully dependent on an increased polyamine pool [76, 77, 82]. A recent finding of Elmarsafawi et al. suggests that glutamine is the primary carbon source for polyamines in antigen-activated effector CD8^+^ T-cells [83]. These data indicate that different subpopulations of immune cells vary in their preferred carbon source for polyamine biosynthesis, thereby competing with tumor cells for both major polyamine precursors. Puleston et al. have shown that polyamines control helper T-cell differentiation and that ODC deficiency results in an inability for CD4^+^ T-cells to adopt correct lineage [78]. Polyamines may also regulate the function of Th1 cells by inhibiting IL-12 production resulting in a significant reduction in IFN-γ production and antitumor functions of helper T-cells [84].

Increased polyamine production is involved in the survival and effector functions of CD8^+^ cytotoxic T-cells [85]. Increased polyamine production by tumors is linked with decreased IL-12 levels and chemokine expression, suggesting that polyamines can both inhibit the effector functions of CD8^+^ T-cells as well as their infiltration into the TME [69, 86, 87]. Conversely, expression of semaphorin 4A (Sema4A) on tumors cells activates mTORC1-mediated polyamine synthesis to support the proliferation of CD8^+^ T-cells without producing an exhaustive phenotype [88]. These results support the hypothesis that increased polyamine synthesis or uptake expressly in T-cells can help support T-cell function and decrease immunosuppression and tumor cell immune evasion. In the context of hepatocellular carcinoma, spermine exerts an immunosuppressive role by elevating *N*-glycosylation and expression of PD-L1 through Akt-dependent β-catenin stabilization [20]. This upregulation of PD-L1 encourages immune evasion by the cancer cells and decreases efficacy of immunotherapies. Polyamines are also indirectly linked to CD8^+^ T-cell function through their regulation of Treg cells. Within the TME, Treg cells contribute to the immunosuppressive environment predominately through inhibition of antigen-presenting cells and secretion of pro-inflammatory cytokines [89]. Spermidine in the TME can enhance the development of naïve T-cells into Tregs leading to an increase in the proportion of immunosuppressive TILs [90].

### Myeloid cells

Tumor-associated myeloid cells (TAMCs) are the most abundant immune cells in the TME of solid tumors and are represented by two main populations: tumor-associated macrophages (TAMs) and myeloid-derived suppressor cells (MDSCs) [91]. Both of these populations, while heterogenous in function and phenotype, support an immunosuppressive microenvironment that is tumor permissive.

Macrophages are essential phagocytic members of the innate immune system. Macrophages serve a variety of roles including phagocytosis, regulation of angiogenesis, antigen presentation, and modulation of inflammation through cytokine secretion [92–94]. The differentiation of macrophages is determined by the cytokines and growth factors present in the tissues in which they infiltrate [95, 96]. Macrophages are recruited to the TME and differentiate into TAMs with their phenotype being a response to the molecules present throughout the TME [91]. Numerous macrophage phenotypes have been described; however, macrophages are broadly classified into two categories: classically activated (M1) macrophages and alternately activated (M2) macrophages. Due to their plasticity, the polarization state of macrophages is fluid and changes in response to its environment [92]. M1 macrophages are pro-inflammatory with a high capacity for antigen presentation and immune activation, while M2 macrophages are considered anti-inflammatory with a poor capacity for antigen presentation. M2 macrophages are often referred to as tumor promoting, as they promote cell proliferation, invasion, and angiogenesis [92, 93, 95]. M2 macrophages also secrete immunosuppressive molecules into the TME and can interact directly with MDSCs to suppress T-cell anti-tumor responses [97].

Central to macrophage polarization is arginine metabolism. M1 macrophages are typically identified as having high NOS activity and low arginase activity, resulting in arginine being preferentially metabolized to NO and citrulline [98]. While NO can be a double-edged sword in cancer biology, it is required for pro-inflammatory macrophage polarization and can result in NO-mediated apoptosis of tumor cells [99]. M1 macrophages can also trigger the activity of natural killer (NK) cells and prime cytotoxic T-cells. M2 macrophages exhibit high arginase activity and low NOS activity, resulting in arginine being fed into polyamine biosynthesis through de novo ornithine synthesis. This promotes an anti-inflammatory phenotype linked to increased angiogenesis, recruitment of MDSCs and regulatory T-cells through chemokine production, and increased expression of both PD-L1 and cytotoxic T-lymphocyte antigen 4 (CTLA4), leading to a decrease in the tumoricidal ability of cytotoxic T-cells [100–102]. Additionally, M2 macrophages can promote invasion and angiogenesis by expressing vascular endothelial growth factor (VEGF) and matrix metalloproteinases (MMPs) [91]. Within the TME, most TAMs are M2 macrophages, which are associated with tumor progression, poor prognosis, and resistance to PD-1 blockade [103].

Polyamines have been implicated in regulating macrophage polarization, particularly in the TME. Putrescine inhibits M1 macrophage activation by downregulating IL-8, while spermidine has been shown to inhibit M1 macrophages by the reduction of pro-inflammatory cytokines and costimulatory molecules CD80 and CD86 [104, 105]. Spermidine also upregulates arginase expression, promoting an M2 phenotype, while spermine can inhibit NOS expression, preventing an M1 phenotype [106, 107]. Mai et al. have demonstrated that expression of IL-33 in esophageal squamous cell carcinoma pushes macrophages toward an M2 polarization by upregulating ODC activity and polyamine biosynthesis [108]. Glioblastoma is characterized by an acidic tumor microenvironment as well as an extensive infiltration of immunosuppressive TAMCs [109]. TAMCs upregulate de novo polyamine synthesis with arginine as the carbon donor. The alkalinity of polyamines buffered the intracellular pH of TAMCs to support their survival in the harshly acidic environment of the solid tumor [109].

MDSCs are a heterogenous population of immature myeloid cells of two primary subtypes: monocytic (M-MDSC) and granulocytic (G-MDSC). Due to their heterogeneity, the molecular phenotypic definition of MDSCs is still controversial and evolving, with G-MDSCs more phenotypically similar to neutrophils and M-MDSCs more similar to macrophages. While a key feature used to define MDSCs is their immunosuppressive nature, M-MDSCs are more immunosuppressive than their G-MDSC counterparts [110]. Similar to M2 macrophages, MDSCs upregulate arginase activity to drive polyamine synthesis. Polyamines then support the growth and immunosuppressive function of MDSCs [111–113]. Elevated polyamine levels also increase the expression of indoleamine 2,3-dioxygenase (IDO1), an enzyme responsible for tryptophan degradation [114, 115]. Metabolites of tryptophan, including kynurenine, inhibit T-cells by inhibiting receptor activation and inducing apoptosis.

### Cancer-associated fibroblasts

Cancer-associated fibroblasts (CAFs) are a heterogenous population of mesenchymal cells that are known to be present in the tumor microenvironment of many solid tumors. They increase tumor tissue stiffness and promote the invasion of cancer cells. CAFs are the main cell type involved in dysregulated collagen turnover and secrete an over-abundance of collagen, which is then crosslinked to increase stiffness within the tumor [116]. The availability of proline is a primary determinant in the ability of CAFs to synthesize and secrete collagen. Proline is synthesized preferentially from P5C, which comes from glutamine and ornithine. Metabolomic analysis of adipose-derived CAFs discovered that polyamines, most notably putrescine, are elevated in CAFs when compared to their mesenchymal stem cell progenitors [117].

CAFs with elevated polyamine biosynthesis also have increased levels of ornithine available for proline and collagen synthesis [118]. High expression of Discoidin Domain Receptor 2 (DDR2) promotes collagen production in both human and mouse omental CAFs. This collagen production is linked to ovarian tumor progression and occurs through increased arginase activity and polyamine production [119]. In pancreatic cancers, arginase-expressing CAFs were found to be a predictor of poorer overall survival. These CAFs expressed very low levels of NOS, indicating that arginine is being preferentially used to generate polyamines through arginase activity [120]. As these CAFs were mostly seen in hypoxic areas, the authors hypothesize that these CAFs are producing extensive proline for collagen synthesis to promote fibrosis of the TME [120, 121]. In non-small cell lung cancer, high arginase expression by CAFs is inversely related to TIL density and is associated with poorer prognosis [122]. Alternatively, polyamines can support invasiveness of transformed fibroblasts by increasing expression of matrix metalloproteinases (MMPs). Kubota and colleagues showed that overexpression of ODC increases invasiveness of mouse fibroblasts both in vitro and in vivo by causing an increase in MMP2, a degrader of collagen type IV [123].

### Endothelial cells

Angiogenesis is the formation of new blood vessels through the migration and growth of endothelial cells. Tumors require neovasculature to provide blood and nutrients as well as a means of transport for metastatic cells. Polyamines are crucial for angiogenesis as they are required for endothelial cell proliferation [124]. Both the arginase and OAT pathways provide ornithine for polyamine synthesis in endothelial cells [125]. Inhibition of polyamine synthesis has been shown to inhibit angiogenesis in tumor models indicating that polyamines support angiogenesis by promoting the proliferation of endothelial cells [126, 127]. Notably, tumors that overexpress ODC produce highly vascularized tumors in mice and inhibition of ODC decreases vasculature independent of VEGF [10, 128]. However, polyamine supplementation has been shown to upregulate the expression of VEGF and various MMPs in vitro [129]*.* Spermidine supplementation, in particular, improves the angiogenic capacity of senescent endothelial cells and enhances ischemia-induced angiogenesis in vivo likely due to an increase in endothelial cell autophagy [130]. Taken together, these data indicate that increased polyamine levels increase the angiogenic capacity of endothelial cells and can lead to increased metastatic ability of tumors.

## Mechanisms of TME immunosuppression by polyamines

### Arginine competition

Polyamines support the survival and function not only of tumor cells but of all cells in the TME. Therefore, the balance and availability of polyamines and their precursors is paramount to the health of the TME. Arginine is a semi-essential amino acid that is utilized in both tumor-suppressive and tumor-permissive functions. Catabolism of arginine by arginase in T-cells promotes their proliferation and activation and can bolster an anti-tumor immune response. CD8^+^ T-cells that preferentially upregulate arginase 1 activity have better effector function due to the sustained production of ornithine and polyamines [131, 132]. Arginine can be metabolized by NOS in M1 macrophages to produce nitric oxide, a signaling molecule required for the release of pro-inflammatory cytokines including IL-1β, TNFα, and IL-17A [133, 134].

**Fig. 2:**
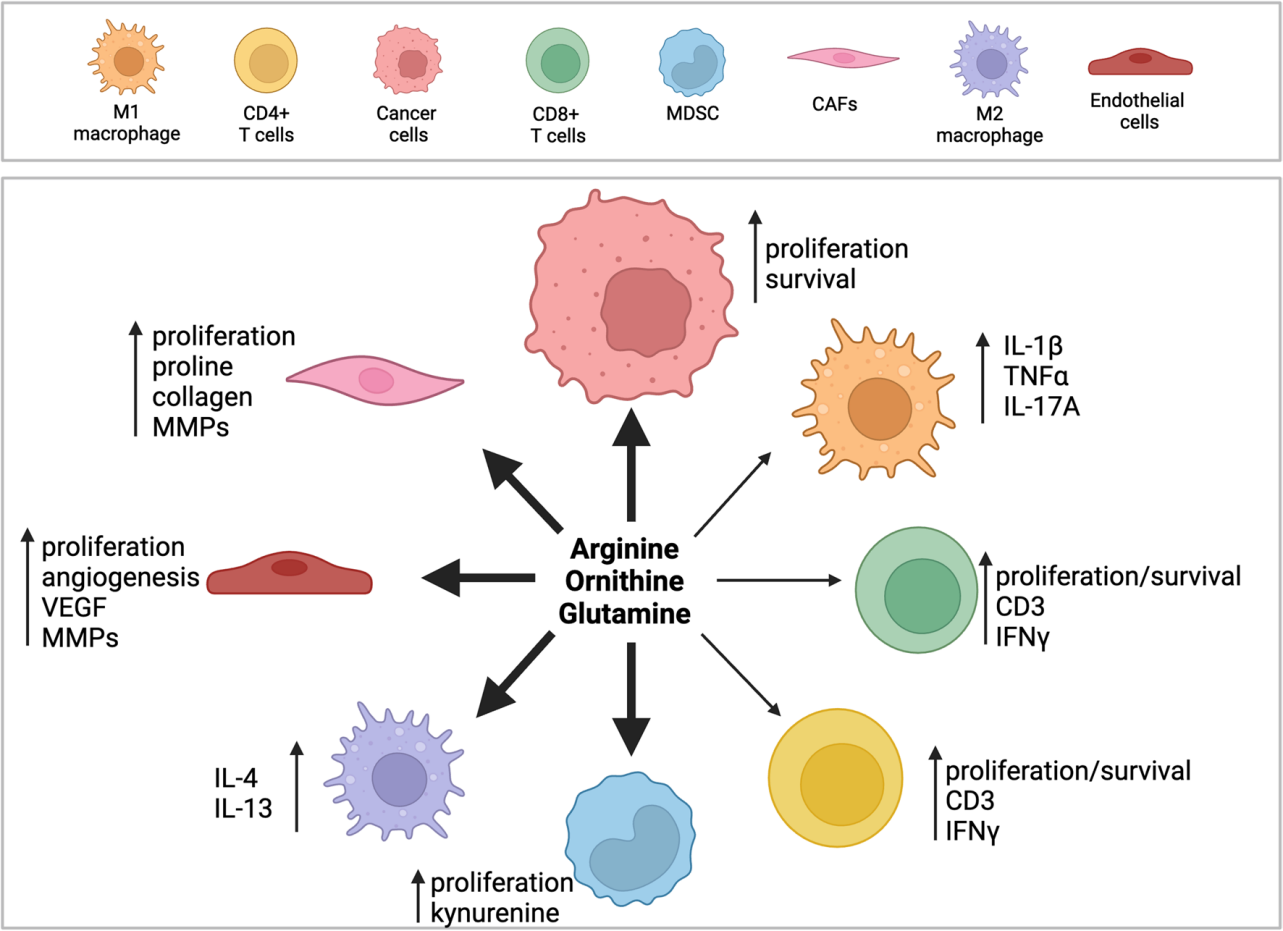
Cells within the TME compete for available amino acids including arginine, ornithine, and glutamine. Amino acids support the proliferation, survival and activity of T-cells in the TME. Pro-inflammatory cytokines are secreted by M1 macrophages following arginine metabolism to nitric oxide. The uptake of amino acids in these cells is severely limited, however, due to competition from tumor and immunosuppressive cells. Tumor cells upregulate arginine, ornithine and glutamine uptake to support their polyamine pool and increase proliferation and survival. Cancer-associated fibroblasts (CAFs) use polyamines to support their deposition of extracellular matrix (ECM) by increasing proline and collagen synthesis as well as increasing matrix metalloproteinases (MMPs) to promote ECM remodeling. Endothelial cells upregulate amino acid and polyamine metabolism to support angiogenesis by increasing proliferation and expression of VEGF and MMPs. The proliferation and function of MDSCs and M2 macrophages are also dependent on polyamine synthesis. Tumor-promoting and immunosuppressive cells preferentially import arginine, ornithine and glutamine to increase polyamine synthesis and support their function thereby depleting pro-inflammatory cells of vital nutrients. Figure created using BioRender.com

In order to persist in the TME, proinflammatory CD4^+^ T-cells, CD8^+^ T-cells, and M1 macrophages must maintain adequate arginine concentrations to support their polyamine needs [135]. There are, however, far more immunosuppressive cells in the TME that require arginine for their function, and T-cells are in direct competition for available arginine (Fig. 2). Arginine is taken up by TAMs and MDSCs, due to their high expression of arginase, resulting in T-cell impairment due to depletion of environmental arginine [136]. This depletion suppresses maturation of the CD3 chain on T cells, making them unable to interact with cancer antigens [137]. In response to IL-4 and IL-10 from tumor cells, M2 macrophages upregulate arginase to metabolize arginine into ornithine [138, 139]. This leads to a positive feedback loop in which M2 macrophages synthesize, release and then re-import polyamines that support their growth and release of immunosuppressive cytokines IL-4 and IL-13. Additional cell types, including tumor cells, CAFs and endothelial cells, also compete with T-cells for arginine, resulting in decreased availability. Overall, arginine within the TME is taken up by tumor cells, MDSCs, M2 macrophages, CAFs, and endothelial cells to be used for polyamine synthesis and support the immunosuppressive and pro-tumorigenic phenotypes of these cells. This results in an immunosuppressive microenvironment, as limited arginine is available in the TME to support the functions of pro-inflammatory cell types such as M1 macrophages and CD4^+^ and CD8^+^ T cells.

### Glutamine competition

Similarly, both tumor cells and immune cells rely on glutamine to sustain survival, homeostasis, and function. Pro-inflammatory immune cells, particularly T-cells, require glutamine for normal function. Glutamine, while usually a minority carbon source for polyamine synthesis, is required for T-cell activation downstream of TCR signaling events [81]. In a glutamine-depleted environment, activated T-cells are less effective, with decreased production of IFN-γ and TNFα [140]. As T-cells are in direct competition with other cells for available glutamine within the TME, it is not uncommon for TILs to have less than optimal glutamine availability.

Many types of tumors exhibit glutamine addiction, where the cell predominately relies on exogenous glutamine [141–144]. Many of the tumors that exhibit glutamine addiction also have aberrant c-myc expression and likely upregulate polyamine biosynthesis [143, 145]. Glutamine is also required for the function of certain immune cells. Oh et al. have shown that glutamine availability is required for MDSC generation and recruitment [146]. Blocking glutamine metabolism led to MDSC cell death and conversion to M1-type macrophages as well as a decrease in IDO expression and kynurenine levels, leading to enhancement of T-cell function [146]. Endothelial cells also require glutamine as a source for the polyamines necessary for proliferation and support of angiogenesis [125]. As previously mentioned, pancreatic cancers use glutamine as a primary source of carbon for polyamine synthesis and therefore significantly upregulate their uptake of glutamine from the TME [55]. This supports tumor cell viability while also reducing the available glutamine for T-cell activation and helps to encourage an immunosuppressive environment.

### T-cell exhaustion

The anti-tumor effects of T-cells are tightly linked to the production of polyamines as well as the metabolism of amino acids including arginine, tryptophan, and methionine. The methionine cycle is intrinsically linked with the polyamine pathway as SAM is decarboxylated to provide the aminopropyl donor for polyamine biosynthesis. Environments where there is aberrantly upregulated polyamine biosynthesis, such as the TME, can lead to a depletion of available methionine. Tumors have also been shown to outcompete T-cells for available methionine by upregulating their methionine transporter [147]. This can lead to decreased anti-tumor immunity from T-cells, as sustained methionine uptake is required for the activation of T-cells and SAM is required for T-cell survival [148].

The term exhaustion is used to refer to T-cells that express inhibitory surface molecules and have a reduced inflammatory capacity. T-cell exhaustion most frequently arises during chronic infections and cancer. Tumor cells can drive T-cell exhaustion by manipulating the methionine cycle of T-cells through competition for available methionine [149]. Following depletion of methionine from the TME, T-cells undergo a global decrease in H3K79me2 and take on an exhausted phenotype including reductions in IFN-γ and granzyme B [147]. Depletion of polyamine levels within T-cells is likely due to competition for uptake with other cells in the TME and is linked with exhaustion phenotypes. Blocking the ability of tumors to uptake extracellular polyamines has shown an increase in immune function and a decrease in exhaustive T-cell phenotypes [21, 150–152].

## Preclinical and clinical relevance of polyamine-based therapies in the TME

### DFMO as a polyamine and immunomodulating therapy in cancer

Difluoromethylornithine (DFMO) is an irreversible inhibitor of ODC that has FDA-approval for the treatment of African trypanosomiasis and as maintenance therapy for high-risk neuroblastoma. DFMO is notably well-tolerated in patients and has been of clinical interest in cancer treatment, prevention and maintenance for decades. More recently, the field has shifted to focus on the immunomodulatory effects of DFMO on the TME of various cancer types.

**Fig. 3:**
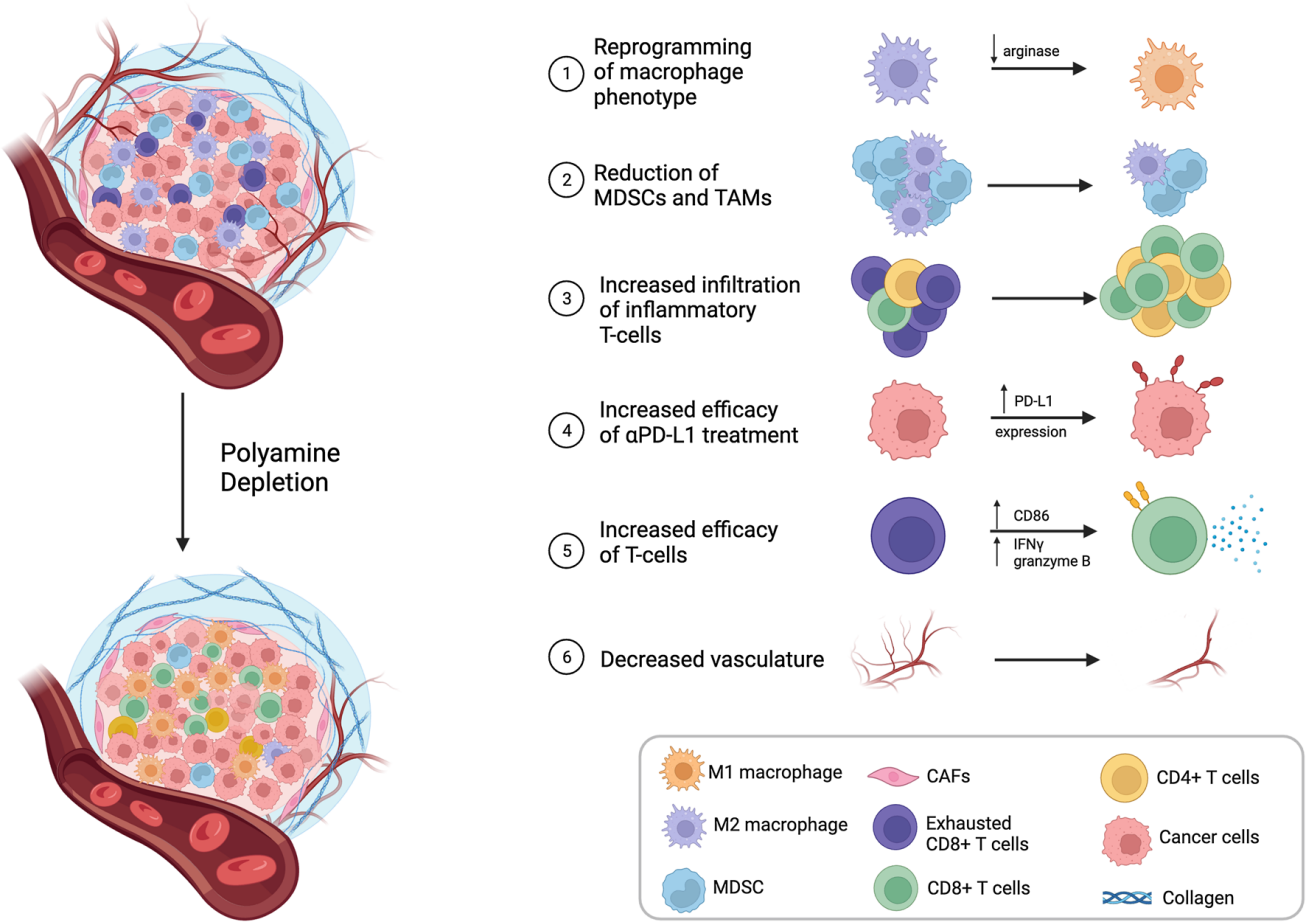
Influence of polyamine depletion on the tumor microenvironment. Depletion of polyamines from immunosuppressive tumor microenvironments can reprogram the microenvironment to a more immune-permissive phenotype. DFMO-mediated depletion of polyamines has been shown to repolarize immunosuppressive M2 macrophages into a more pro-inflammatory M1 phenotype (1). Polyamine depletion has also been shown to reduce MDSCs and TAMs while increasing the infiltration of inflammatory T-cells into the TME (2, 3). Efficacy of T-cells can be increased by TME polyamine depletion resulting in a decrease of exhausted T-cell phenotypes and increased PD-L1 expression on tumor cells (4, 5). Lastly, DFMO-treated tumors exhibit less neovasculature than untreated tumors indicating that polyamine depletion may be protective against metastasis [10, 126]. Figure created using BioRender.com

In a murine model of glioblastoma, DFMO treatment increases survival, reduces polyamines, and is sufficient to reduce immunosuppression in the TME [109]. There was significant reduction in MDSCs and TAMs with DFMO treatment. Importantly, the antitumor immunity driven by DFMO is dependent on T-cells, and treatment induces a decrease in the myeloid-to-T-cell ratio in the TME. DFMO treatment also decreases arginase expression in the monocyte compartment, perturbing the arginine metabolic pathway and encouraging a reprogramming of M2 macrophages into more pro-inflammatory M1 macrophages (Fig. 3) [109]. DFMO treatment upregulates PD-L1 expression levels of tumors both in vitro and in vivo. Impressively, therapy combining DFMO and anti-PD-L1 treatment has an additive benefit on survival of the CT-2A model, which is usually resistant to checkpoint blockade [109]. These data suggest that in glioblastoma, DFMO is sufficient to blunt TAMC-induced TME immunosuppression and sensitize the tumor to immune checkpoint blockade.

DFMO has shown limited clinical efficacy as a single agent in cancer treatment due to cancer cells compensating for decreased polyamine biosynthesis by upregulating polyamine transport [153]. While the polyamine transport system in mammals is not as well-defined as in other organisms, recent work has shed some light onto some of the likely mechanisms involved. Potential mechanisms have implicated both polyamine permeases and receptor-mediated endocytosis as a cellular entry point for polyamines [154–156]. Intracellular polyamines are found in polyamine-sequestering vesicles (PSVs) from which they can be released into the cytoplasm. Numerous P5B-type ATPases have been recently implicated in mammalian polyamine transport including ATP13A2, ATP13A3, and SLC18B1 [157–164]. Notably, no individual proposed mechanism accounts for all biochemical data available indicating that mammalian polyamine transport likely occurs by multiple mechanisms.

DFMO treatment inhibits polyamine biosynthesis but causes the compensatory upregulation of the polyamine transport system [165, 166]. A strategy termed polyamine blocking therapy (PBT) leverages the combination of DFMO with a polyamine transport inhibitor to bypass the transport compensation of cancer cells. The polyamine transport inhibitor, AMXT 1501, combined with DFMO significantly reduces polyamine levels and inhibits the growth of numerous tumors types in vivo including melanoma, colon, and mammary adenocarcinoma [85, 150, 152]. Notably, nude mice do not respond to PBT, and PBT treatment in a syngeneic mammary adenocarcinoma model prevented rechallenge, indicating that the immune system is central to the PBT response and likely promotes immune memory [85, 152]. PBT using Trimer44NMe as the polyamine transport inhibitor has also produced encouraging preclinical data. The combination of DFMO with Trimer44NMe decreases tumor burden and increases survival in gemcitabine-resistant pancreatic cancer murine model systems [167, 168]. Nakkina et al. have shown that PBT increases the expression of CD86, a T-cell co-stimulatory marker, and causes a nearly threefold increase in M1 macrophage infiltration into the TME [19]. Alexander et al. evaluated the efficacy of PBT in colon cancer and found that PBT therapy increased CD8^+^ T-cells and their activity as indicated through increased IFNγ and granzyme B expression [150]. The authors also noted a decrease in M2 macrophages, MDSCs and Tregs [150]. Subsequent work indicated that general control nonderepressible 2 (GCN2), a sensor that detects amino acid depletion, is required for the anti-tumor efficacy of PBT. These data indicate that GCN2 activation in response to increased polyamine synthesis and arginine depletion promotes immunosuppression in the TME through protection of MDSCs and M2 macrophages [169].

DFMO treatment has been shown to enhance α-PD-1 checkpoint blockade in both susceptible and refractory cancer models. Dryja et al. tested DFMO in combination with α-PD-1 therapy in Lewis lung carcinomas and PD-1 blockade-resistant B16F10 melanoma tumors [21]. DFMO and α-PD-1 combination therapy synergistically improved overall survival, with multiple complete responders, and enhanced the survival and activity of tumor-infiltrating CD8^+^ T-cells (Fig. 3). Alexander et al. showed that the combined administration of lower-dose DFMO and the Trimer44NMe polyamine transport inhibitor enhanced the sensitivity of 4T1 mammary and B16F10 melanoma tumors to PD-1 blockade [151]. In addition to increasing the survival of mice, treatment increased tumor-specific CD8+ T-cells and decreased tumor infiltrating immunosuppressive myeloid cells (Fig. 3). Additionally, DFMO-treated pancreatic tumors exhibit an increase in CD4^+^ T-cell infiltration [19]. These data support the hypothesis that DFMO can enhance the response of cold tumors to checkpoint blockades.

DFMO has the potential to be combined with drugs outside of polyamine metabolism. Travers et al. combined DFMO treatment with the DNA methyltransferase inhibitor 5-azacytidine (AZA) in the VDID8^+^ murine ovarian cancer model, resulting in increased survival and immune modulation [18]. Treatment increased NK cells and IFNγ+ T-cells in ascites fluid while simultaneously decreasing CD11b+ macrophages [18]. Interestingly, these changes did not increase the model’s response to α-PD-1 therapy, indicating the T-cell response was not the primary mechanism driving the survival benefit in this model. The authors discovered that depletion of M1 macrophages eliminated the survival benefit of the combination DFMO/AZA treatment and postulated that it exerts its effects by repolarizing M2 macrophages into the M1 phenotype [18].

While the currently available immunological data were mostly obtained using DFMO as the means for polyamine depletion, it is quite possible that other mechanisms of polyamine depletion in the TME would prove effective in reducing TME immunosuppression. Current compounds of interest include ivospemin (SBP-101), a symmetrically substituted spermine analogue that competes with natural polyamines for uptake [170–172]. Ivospemin decreases polyamine levels in vitro by decreasing ODC activity and increasing activity of the polyamine catabolic enzyme SSAT [173]. Holbert et al. discovered that ivospemin treatment of the VDID8^+^ syngeneic mouse ovarian model decreased tumor burden and increased overall survival with decreased polyamine levels observed in the ascites fluid [173]. Additionally, ivospemin has demonstrated anti-tumor efficacy in pancreatic cancer in vitro and in vivo and is currently enrolling a Phase 2/3 trial evaluating its efficacy in combination with gemcitabine and nab-paclitaxel in patients with metastatic pancreatic cancer [170, 171, 174] (Table 1). The influence of ivospemin on the TME warrants further investigation as it is likely that its inhibition of ODC increases available arginine for T-cells in the TME and decreases available polyamines for immunosuppressive cell types.

**Table 1:** Current clinical trials employing polyamine depletion strategies

Polyamine-based treatment	Combination drugs	Disease	Current phase	ID
DFMO	Etoposide	Neuroblastoma (relapsed/refractory)	Phase 2	NCT0401943 NCT01059071
DFMO and AMXT 1501		Advanced solid tumors	Phase 1/2	NCT05500508 NCT03536728
DFMO and AMXT 1501		High grade glioma	Phase 1	NCT05717153
DFMO		Neuroblastoma	Phase 2	NCT02679144
DFMO	Temozolomide	Glioblastoma	Phase 1b	NCT05879367
DFMO	Irinotecan, temozolomide and dinutuximab	Neuroblastoma (relapsed/refractory)	Phase 2	NCT03794349
DFMO	High dose testosterone with enzalutamide	Prostate cancer	Phase 2	NCT06059118
DFMO		High risk neuroblastoma (in remission)	Phase 3 FDA approved	NCT02395666
Ivospemin (SBP-101)	Nab-paclitaxel and gemcitabine	Pancreatic	Phase 2/3	NCT05254171 NCT03412799 NCT0265733

Polyamine depletion via DFMO treatment is being clinically tested in a variety of cancers including neuroblastoma, glioma, glioblastoma and prostate cancer. Additionally, the polyamine analogue ivospemin is in phase 3 clinical trials for metastatic pancreatic cancer

### Polyamine metabolism gene expression profiles as prognostic markers for immunotherapy response

Polyamine metabolism gene expression can also be used as a prognostic indicator for response to immunotherapy. Leveraging immunogenic T-cell-infiltrated, HPV-related head and neck cancers, Harbison et al. identified upregulation of polyamine synthesis and metabolism-related genes as a poor prognostic indicator. High expression of polyamine-related genes was associated with aggressive molecular phenotypes, poor prognosis, diminished antitumor immunity and poor response to immunotherapy [175]. High levels of polyamines in epithelial ovarian cancer are also associated with decreased cancer immunity [176]. Colorectal cancer patients with high expression of polyamine metabolism genes were associated with more advanced stage, higher infiltration levels of immunosuppressive cells and unfavorable prognosis [22]. High polyamine metabolism in these patients was also associated with microsatellite stability, low mutational burden, and unfavorable response to immunotherapies [22]. High levels of polyamine metabolism are also a marker for poorer prognosis in clear cell renal carcinoma. Surprisingly, Chen et al. found that clear cell renal carcinoma patients with high expression of polyamine-related genes show an increase in immune infiltrate but a poorer response to immunotherapies [177]. The authors discovered that high expression of polyamine-related genes was associated with increased Tregs in the TME and increased expression of T-cell exhaustion markers such as CTLA-4, TIGIT, and LAG3, indicating that while there may be more immune infiltrate in the TME, they may not be sensitive to immunotherapy due to immune escape [177]. It is important to note that while gene expression of polyamine metabolism-related genes may be of prognostic value, it is not necessarily informative of polyamine levels in the tumor.

### Polyamines as cancer biomarkers

Immunologically cold cancers often exhibit extremely high levels of polyamine metabolism. While the idea of polyamines as biological markers in cancer is decades old, recent work has provided further evidence that polyamines may be a viable biomarker for cancer diagnosis including in numerous immunologically cold tumor types [178]. Polyamine metabolite levels in the blood have been proposed as a biomarker for the early detection of ovarian cancer [179]. Significantly decreased urinary spermine was observed by Tsoi et al. in prostate cancer samples compared to healthy controls, however spermine levels were not significantly correlated with Gleason grade [180]. Urinary acetylated polyamines, particularly diacetylated spermine, have potential as tumor markers for breast and colon cancer [181]. Additionally, higher levels of polyamines are found in aggressive subtypes of epithelial ovarian cancer such as endometroid carcinoma and high-grade serous ovarian carcinoma [182].

### Clinical polyamine modulating therapies

There are numerous clinical trials investigating the potential of DFMO as part of a combinatorial strategy in cancer, and additional trials investigating DFMO as a maintenance or chemopreventative strategy (Table 1). DFMO treatment is well studied in neuroblastoma and glioblastoma [183, 184]. In December 2023, DFMO had its first FDA approval as a cancer therapeutic for pediatric patients with high risk neuroblastoma who are in remission (NCT02395666). Other polyamine-modulating drugs are currently being investigated in the clinic, including the polyamine transport inhibitor AMXT 1501 and polyamine analogue ivospemin (SBP-101) **(**Table 1**)**. Notably, nearly all current clinical trials modulating polyamine metabolism are being completed in tumor types that are traditionally considered immunologically “cold” such as prostate, brain, and pancreatic cancer.

## Conclusions

Nearly all cells are completely reliant on polyamines for proliferation, function, and survival. Due to increased metabolic needs, polyamines and their precursors are overly abundant in tumors and in the tumor microenvironment. Polyamines help sustain pro-tumorigenic microenvironments by supporting the function and survival of immunosuppressive cells such as TAMs, MDSCs, and Tregs as well as the proliferation of stromal cells including CAFs and endothelial cells. Cells of the TME outcompete pro-inflammatory cells, such as TILs and M1 macrophages, for nutrients and thereby decrease the anti-tumorigenic capacities of these cells. Pharmacological depletion of polyamines can reprogram the TME into a more immune-permissive phenotype by recruiting pro-inflammatory cells, decreasing immunosuppressive cells, and decreasing exhaustion phenotypes in T-cells. Polyamine depletion increases the efficacy of immunotherapy in vivo and may be of particular clinical interest in immunologically cold tumors traditionally unresponsive to immune checkpoint blockade.

## Data Availability

No datasets were generated or analysed during the current study.
